# Omeprazole taken once every other day can effectively prevent aspirin-induced gastrointestinal mucosal damage in rats

**DOI:** 10.1186/s12876-024-03265-0

**Published:** 2024-05-29

**Authors:** Junhua Weng, Yuli Song, Dayu Kuai, Weiwei Dai, Yuxia Yao, Wenjing Xu, Yaqiang Li, Longying Fan, Baohong Xu

**Affiliations:** Department of Gastroenterology, Beijing Lu He Hospital, Capital Medical University, 82 Xinhua South Road, Beijing, 101149 P.R. China

**Keywords:** Low-dose aspirin, Low-frequency omeprazole, Omeprazole once every other day, Hypergastrinemia, Gastrin

## Abstract

**Background:**

Proton-pump inhibitors (PPIs) prevent aspirin-associated gastric and duodenal mucosal damage. However, long-term use of PPIs can lead to various adverse reactions, such as gastric polyps and enterochromaffin-like cell hyperplasia. Current research indicates that the abovementioned adverse reactions are mainly related to hypergastrinemia. We investigated whether low-frequency administration of omeprazole could effectively repair aspirin-induced mucosal damage and reduce the increase in gastrin levels associated with long-term use of PPIs.

**Methods:**

Sprague‒Dawley rats were divided into four treatment groups: daily aspirin, daily aspirin and omeprazole once every day (qd), daily aspirin and omeprazole once every other day (qod), and daily aspirin and omeprazole once every three days (1/d3). After 15 days of feeding, blood samples were collected, and the stomachs of sacrificed rats were subjected to macroscopic, histological, and immunohistochemical studies. Moreover, in clinical practice, patients with peptic ulcers caused by aspirin took a standard dose of omeprazole (20 mg) every other day. Two months later, gastroscopy was performed to examine the healing of the ulcers.

**Results:**

Both the omeprazole qd and omeprazole qod administrations effectively prevented aspirin-induced gastric peptic ulcers, with no significant difference between the two groups in the inhibition of parietal cell secretion of gastric acid and cell apoptosis. However, omeprazole 1/d3 failed to completely prevent aspirin-induced gastric mucosal injury. Notably, the gastrin levels, cell proliferation ability and cholecystokinin B receptor expression of the omeprazole qd group were significantly higher than those of the omeprazole qod group. In clinical work, patients with peptic ulcers caused by aspirin were given a standard dose of omeprazole every other day, and their ulcers healed after 2 months, as observed by gastroscopy.

**Conclusions:**

Omeprazole administration once every other day can effectively prevent aspirin-induced peptic ulcers and reduce hypergastrinemia, which may reduce the long-term adverse effects of PPI treatment.

**Supplementary Information:**

The online version contains supplementary material available at 10.1186/s12876-024-03265-0.

## Introduction

Aspirin, which is a nonsteroidal anti-inflammatory drugs (NSAIDs), is widely used in patients with cerebrovascular, coronary, or peripheral artery disease [1]. However, studies have shown that aspirin increases gastrointestinal (GI) damage by inhibiting prostaglandin synthesis, inducing direct cytotoxicity, and causing microvascular injury. In a prospective study, the cumulative ulcer incidence rate of aspirin users at 12 weeks was 7.3%, and aspirin users with a history of ulcer bleeding had a recurrent bleeding rate of 15% within 1 year [2].

PPIs are indispensable in the treatment of gastritis, peptic ulcers, peptic ulcers with bleeding and other diseases [3]. Currently, long-term use of PPIs is approved to prevent peptic ulcers with bleeding induced by NSAIDs. However, recent studies have shown that long-term use of PPIs can induce gastrin elevation, resulting in hypergastrinemia, fundic gland polyps, intestinal chromaffin cell (ECL) hyperplasia and other adverse reactions [4]. There is a linear relationship between the dose and duration of PPI administration and gastrin elevation [5]. It is presumed that reducing the dose of PPIs may reduce the adverse effects of long-term use of PPIs. Ally MR et al. found that giving half of the normal dose of PPIs to patients with chronic renal disease effectively prevented GI bleeding [6]. Based on the above research, we studied the optimal degree of PPI dose reduction, which can not only effectively prevent NSAIDs-related GI bleeding but also reduce the adverse reactions of long-term use of PPIs.

In this study, a peptic ulcer model in Sprague‒Dawley (SD) rats was induced by aspirin, and the effects of omeprazole (Ome) at different dosing frequencies on ulcer healing and gastrin expression were observed. In addition, in clinical work, patients with aspirin-related peptic ulcers took the standard dose of omeprazole every other day, and ulcer healing was checked by gastroscopy after 2 months.

## Materials and methods

### Animals and treatments

Male SD rats (250–300 g) aged 8 weeks old were purchased from Shanghai SLAC Laboratory Animal Co., Ltd. (Shanghai, China), housed in hygienic cages at 22–24 °C with a 12-h cycle of darkness and light and had free access to water and food. The present experimental study was designed and performed according to the “Animal Research: Reporting of In Vivo Experiments” or ARRIVE guidelines. After a 1-week adaptation period, rats were randomly divided into four groups (*n* = 8 per group) as follows:

#### Asp group

The rats received aspirin (150 mg/kg) through oral gavage once a day [7].

#### Asp + Ome qd group

The rats received aspirin (150 mg/kg) and omeprazole (20 mg/kg) through oral gavage once a day [8].

#### Asp + Ome qod group

The rats received aspirin (150 mg/kg) through oral gavage once a day and received omeprazole (20 mg/kg) through oral gavage once every other day.

#### Asp + Ome 1/d3 group

The rats received aspirin (150 mg/kg) through oral gavage once a day and received omeprazole (20 mg/kg) through oral gavage once every 3 days.

**Fig. 1:**
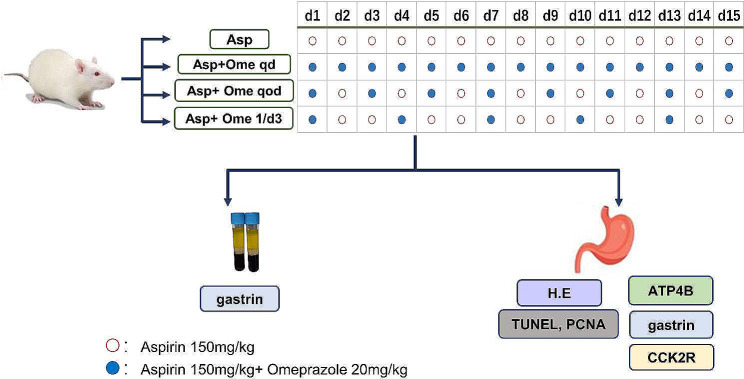
Diagram showing the experimental design. According to different administration methods of omeprazole, SD rats were randomly divided into 4 groups (Asp group: aspirin once a day; Asp + Ome qd group: aspirin and omeprazole once a day; Asp + Ome qod group: aspirin once a day and omeprazole once every other day; Asp + Ome 1/d3 group: aspirin once a day and omeprazole once every 3 days.) to observe the protective effect of different doses of omeprazole on aspirin-induced gastric mucosal injury. Asp: aspirin; Ome: omeprazole

Aspirin (A6810, Sigma Aldrich, St. Louis, MO, USA) and omeprazole (Gartunavagen, Sodertalje, SE-15,185, Sweden, AstraZeneca AB) were suspended in 1% carboxymethylcellulose (CMC) (C5678, Sigma Aldrich, St. Louis, MO, USA). After all animals were randomized, 100 µL of blood was drawn from the tail vein of each rat to detect the content of serum gastrin 17. Then, each rat was given aspirin and/or omeprazole by gavage feeding at 9:00 am for 15 days. At the end of the experiment (day 15), after 10 h of fasting, the rats were sacrificed by carbon dioxide inhalation, and blood samples were collected from the abdominal aorta in time. The serum was separated and stored at -20 °C for further analysis. The stomach was removed after the oesophageal end was tied, and the gastric contents were collected and subsequently centrifuged. Whole stomach tissue was excised for ulcer assessment and biochemical analysis. An overview of the experimental design is shown in Fig. 1.

### pH value of gastric contents

After dissection, the oesophageal end was tied, the stomach was removed, and the gastric contents were collected and centrifuged (3500 rpm, 10 min). The supernatant was used for pH determination. First, a wide range of pH test papers was used to determine the approximate range of pH values, and then precision pH test paper within this range was used for more accurate measurements (Sinopharm Chemical Reagent Co., Ltd., China). The colour of each pH test paper was examined by three people without any known colour vision disorders, and the average value was used as the final pH value of the gastric juice.

### Gross damage and histological index

Whole stomach tissue was harvested and opened along the greater curvature, and the mucosa was exposed for ulcer evaluation. The opened stomach tissue was fixed with needles, and red colouration and haemorrhagic streaks in the ulcerated area were observed. The formation of gastric ulcers was assessed pathologically using both the damage area and ulcer index. The damaged area was assessed using planimetry under 1 mm × 1 mm graph paper with a recording camera and expressed as a percentage of the total area of the stomach.

The gross damage of the gastric mucosa was assessed by two experienced gastroenterologists who were blinded to the experiment using a previously described gross ulcer index defined as (number of type I lesions)+(number of type II lesions)×2+(number of type III lesions)×3 [9]. The lesion type was classified as follows: type I indicated the presence of oedema, hyperaemia, or a single submucosal punctiform haemorrhage; type II was defined as the presence of submucosal haemorrhagic lesions with small erosions; and type III indicated the presence of a deep ulcer with erosions and invasive lesions. A total injury score for each stomach was calculated by summing the gross ulcer index of all lesions in that stomach.

### Immunohistochemical methods

Immunohistochemical staining was performed as previously described [10]. The primary antibodies used were proliferating cell nuclear antigen (PCNA, 1:500, cat. no. 13,110, Cell Signaling Technology, Inc.), H+/K+-ATPase β (ATP4B, 1:200; cat. no. sc-374,094, Santa Cruz Biotechnology, Inc.), gastrin (1:200, cat. no. ab232775, Abcam), and cholecystokinin B receptor (CCK2R, 1:100, cat. no. sc-166,690, Santa Cruz Biotechnology, Inc.). The positively stained areas and positive cells were evaluated using Image-Pro Plus 6.0 software (Media Cybernetics Inc.). Staining was quantified in 10 randomly selected ×200 high-power fields per tissue sample.

### TUNEL assay

To detect cellular apoptosis in stomach tissue, a terminal deoxynucleotidyltransferase (dUTP)-mediated nick end-labelling (TUNEL) assay was performed using an In Situ Cell Death/Apoptosis Detection kit (cat. no. 11,684,817,910; Roche Diagnostics, GmbH) was performed as previously described [10]. The results were scored semiquantitatively by averaging the numbers of TUNEL-positive cells per high-power field (magnification, ×200) for 10 fields per tissue sample with ImageJ software (National Institutes of Health).

### Haematological investigation

Gastrin 17 levels in rat serum were assessed using a commercially available ELISA kit (cat. no. ml059375, Nanjing Jiancheng Bioengineering Institute, China) according to the manufacturer’s protocol based on the quantitative sandwich enzyme immunoassay technique.

### Clinical effects

After accepting and signing the informed consent form, two patients with peptic ulcers who were taking aspirin (100 mg/d) (one patient with coronary atherosclerotic heart disease and another patient with hyperlipidaemia and arteriosclerosis, average age: 55.5 years) were given omeprazole (Losec, AstraZeneca AB, 20 mg/tablet) once every other day (qod). Meanwhile, the patients continued to take aspirin. After 2 months, gastroscopy was reviewed to observe the repair of gastric mucosal injury.

### Statistical analysis

All analyses were performed using IBM SPSS Statistics 27.0 software (IBM Corp, Armonk, NY). Statistical significance was determined using nonparametric statistics, the Mann‒Whitney *U* test, or the Kruskal‒Wallis test. The data are presented as the medians [interquartile ranges (IQRs)]. Differences for which *p* was less than 0.05 were considered statistically significant.

## Results

### Evaluation of gastric lesions

**Fig. 2:**
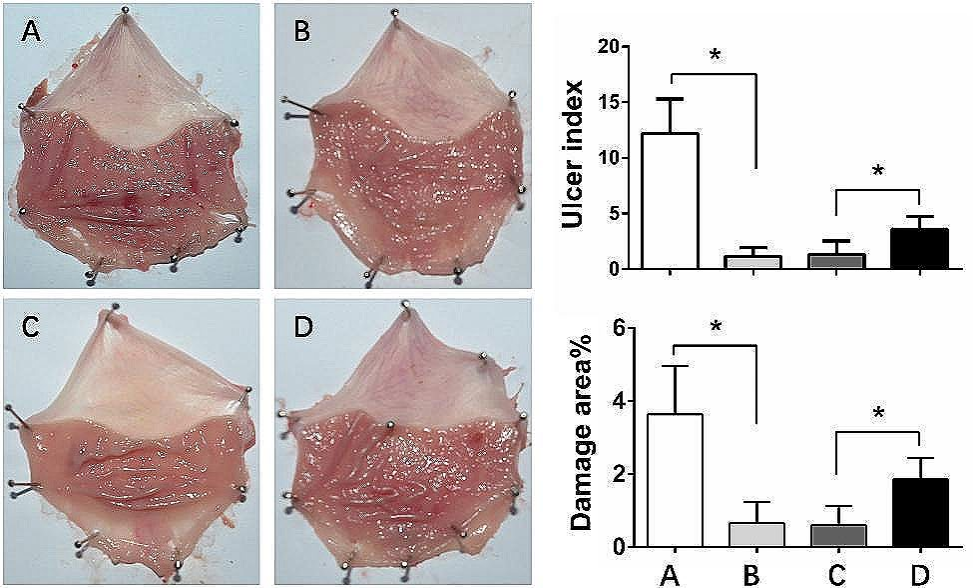
Protective effect of different doses of omeprazole on aspirin-induced gastric ulcers. The stomach of the aspirin group (**A**) exhibited long dark red submucosal haemorrhagic stripes. The stomach of the Asp + Ome qd group (**B**) and Asp + Ome qod group (**C**) predominantly showed pink normal gastric mucosa. In the Asp + Ome 1/d3 group (**D**), scattered submucosal haemorrhage spots and short stripes were observed in the stomach. (*p<0.05, Asp: aspirin, Ome: omeprazole)

The administration of aspirin for 15 days resulted in severe haemorrhagic streaks in the dissected stomach of rats (Fig. 2A), whereas the lesions were repaired by concomitant treatment with omeprazole. However, the intensity of damage repair was closely related to the way omeprazole was administered. The gross damage to the gastric mucosa was assessed by the damage area and ulcer index. No obvious gastric mucosa injury was observed in the groups administered omeprazole once a day and once every other day, and there was no significant difference in the ulcer index or mucosal injury area between the two groups (Fig. 2B and C). The degree of gastric mucosal injury in the groups given omeprazole once every 3 days was significantly less than that in the aspirin group, but small patches of mucosal haemorrhage were still visible (Fig. 2D).

### Acid-inhibiting ability of omeprazole with different administration methods

**Fig. 3:**
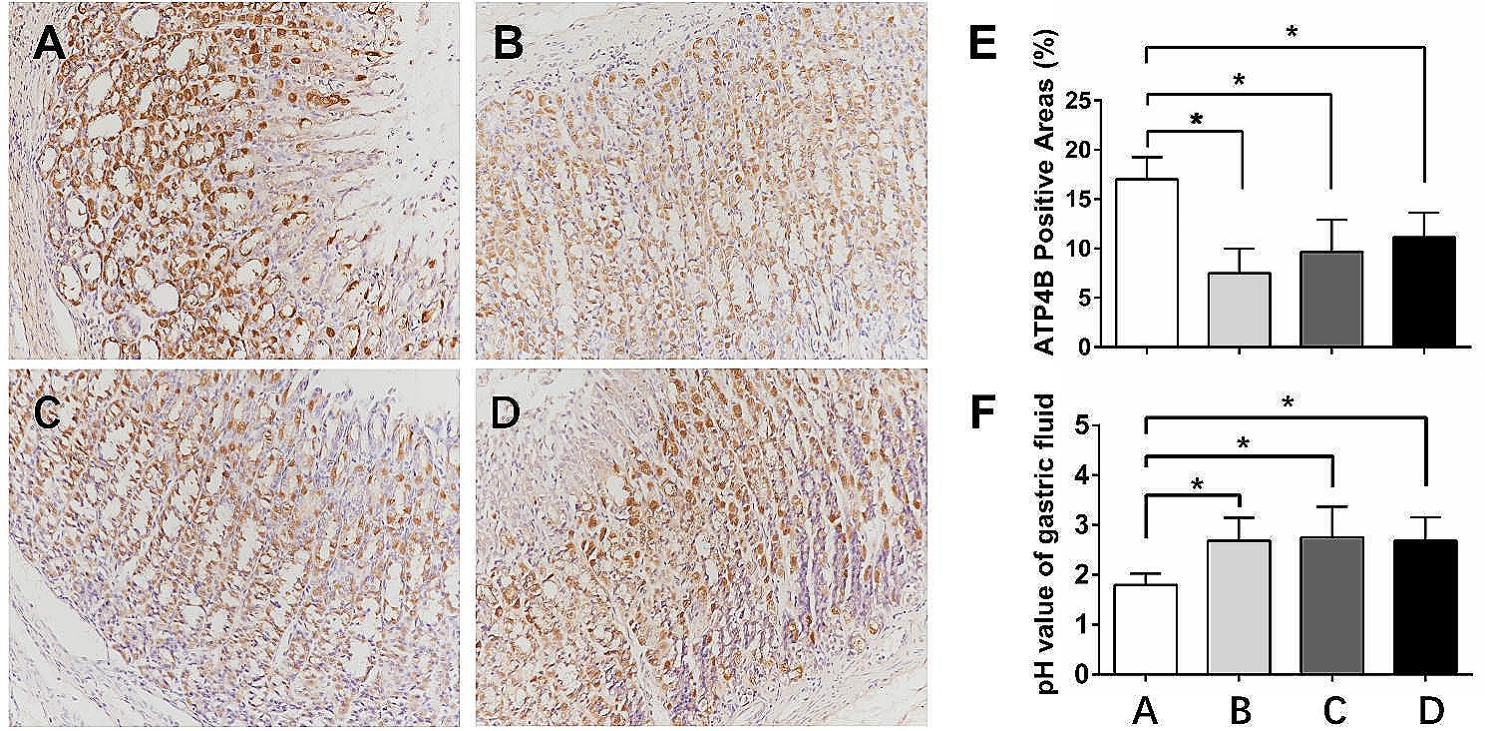
Effect of different doses of omeprazole on gastric acid secretion. The expression of ATP4B in gastric mucosa by immunohistochemical staining (200 × magnification): The expression of ATP4B in the aspirin group (**A**) was significantly higher than that in the Asp + Ome qd group (**B**), Asp + Ome qod group (**C**) and Asp + Ome 1/d3 group (**D**), while there was no significant difference between the latter three groups. (**E**) ATP4B-positive areas. (**F**) The pH value of gastric juice was measured with a precise pH test paper. (*p<0.05, Asp: aspirin, Ome: omeprazole)

Omeprazole reduced gastric acid secretion by inhibiting the H+/K+-ATPase activity of gastric parietal cells. The H^+^/K^+^-ATPase activity in the aspirin group was high (Fig. 3A), while the activity was significantly inhibited in the omeprazole group administered this PPI once a day (Fig. 3B). The activities of H^+^/K^+^-ATPase in the omeprazole group administered this PPI once every other day and the omeprazole group administered the drug once every three days were also inhibited, although the degree of inhibition was lower than that in the omeprazole group treated once a day, but a significant difference was not observed (Fig. 3C and E). We also measured the pH value of gastric juice in rats. The pH of the gastric juice in the aspirin group was approximately 1.8, while the pH of the gastric juice in the three groups taking omeprazole at different frequencies was significantly increased by approximately 2.8, and there was no significant difference in pH values among the three groups (Fig. 3F).

### Proliferation and apoptosis of gastric mucosal cells

**Fig. 4:**
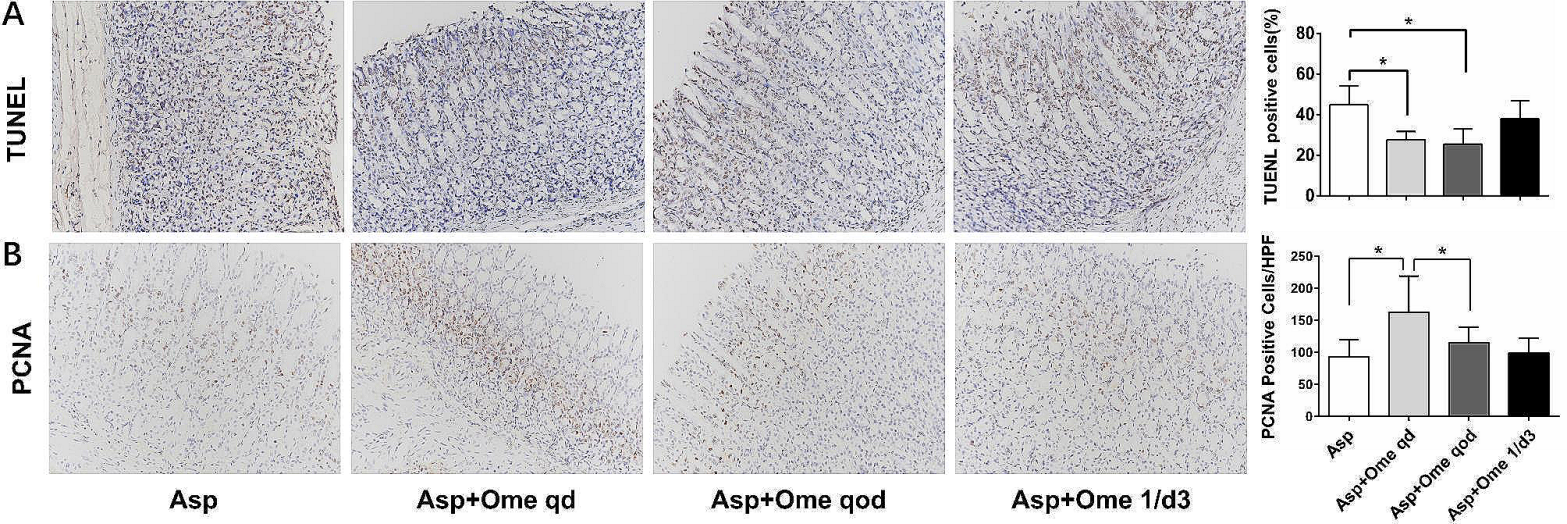
Effects of different doses of omeprazole on apoptosis and proliferation of gastric mucosal cells. (**A**) TUNEL method, (**B**) the expression of PCNA by immunohistochemical staining. (200 × magnification). (*p<0.05, Asp: aspirin, Ome: omeprazole)

Aspirin can lead to an increase in apoptotic cells in the gastric mucosa and a decrease in cell proliferation. Compared with that in the aspirin group, apoptosis in the omeprazole once a day group and the omeprazole once every other day group was significantly reduced, and there was no significant difference between the two groups, while the ability of omeprazole once every three days to reduce apoptosis was weaker than that in the former two groups (Fig. 4A). The cell proliferation ability of the omeprazole once daily group was significantly higher than that of the other two groups (Fig. 4B).

### Low-dose omeprazole reduced gastrin and CCK2R secretion

**Fig. 5:**
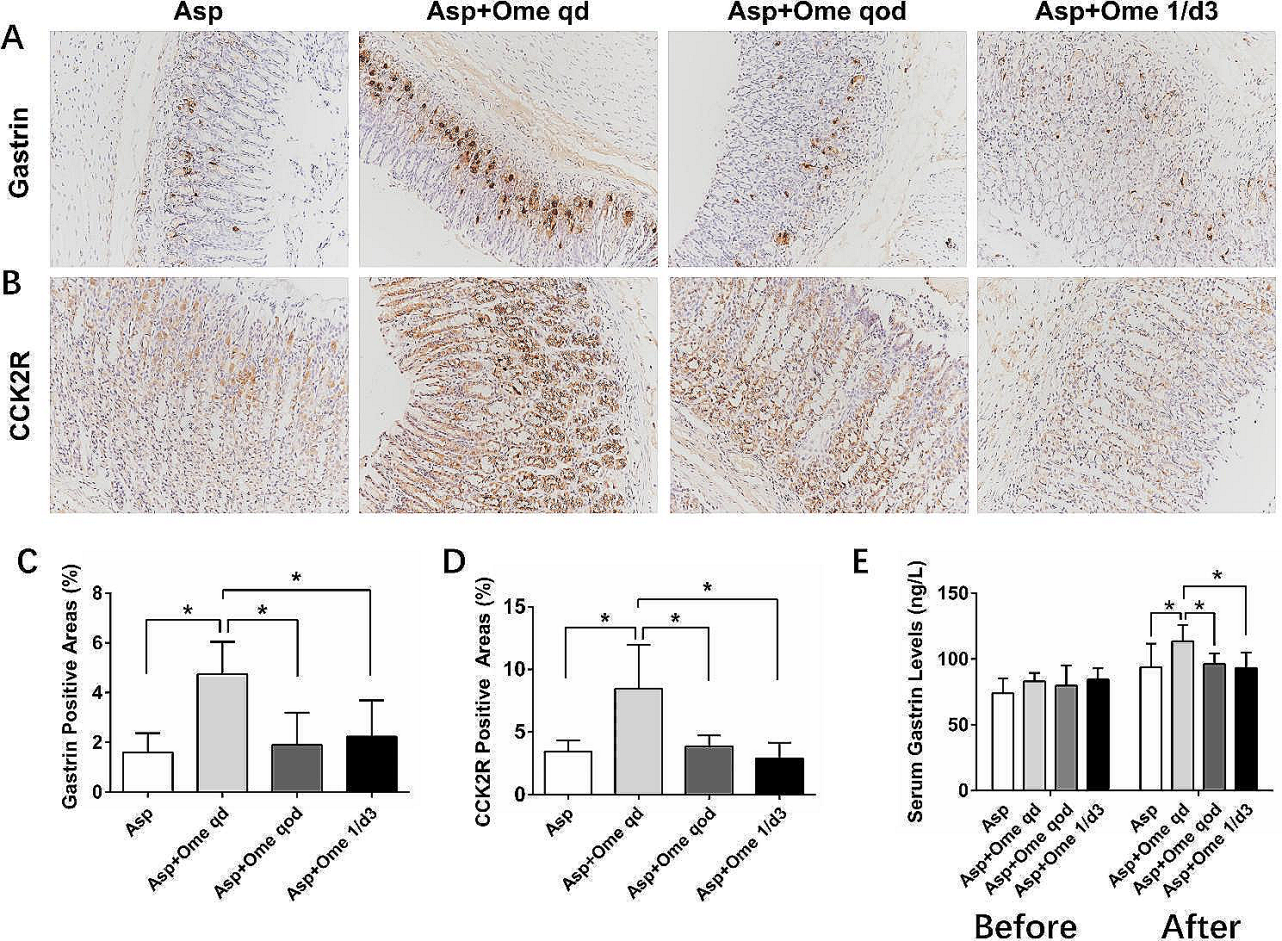
The expression of gastrin (**A**) and CCK2R (**B**) by immunohistochemical staining (200 × magnification). The gastrin-positive (**C**) and CCK2R-positive (**D**) areas. (**E**) The serum gastrin levels before (i.e., at the beginning of the group) and after the experiment. (*p<0.05, Asp: aspirin, Ome: omeprazole)

The secretion of gastrin in the aspirin group was low (gastrin-positive area: 1.44[0.90–1.91]). Omeprazole once a day significantly promoted the secretion of gastrin (4.11[3.56–4.67]). The stimulatory effect of omeprazole once every other day and once every three days on gastrin was significantly weaker than that of omeprazole once a day, and there was no difference in gastrin levels between the two groups (2.11[1.15–2.55] vs. 1.14[0.92–2.45], *p* > 0.05) (Fig. 5A and C). Moreover, the gastrin secretion levels of the two groups were basically comparable to those of the aspirin group, with no significant increase. The serological results were consistent with the histological results (Fig. 5E).

CCK2R, as a receptor for gastrin, is mainly expressed in gastric ECL cells and parietal cells. The expression trend of CCK2R was consistent with that of gastrin, and the expression of CCK2R stimulated by omeprazole once a day was significantly stronger than that stimulated by omeprazole once every other day (9.32[6.60-10.28] vs. 3.15[2.80–4.10], *p* < 0.05) (Fig. 5B and D).

### Omeprazole taken once every other day effectively healed peptic ulcers

**Fig. 6:**
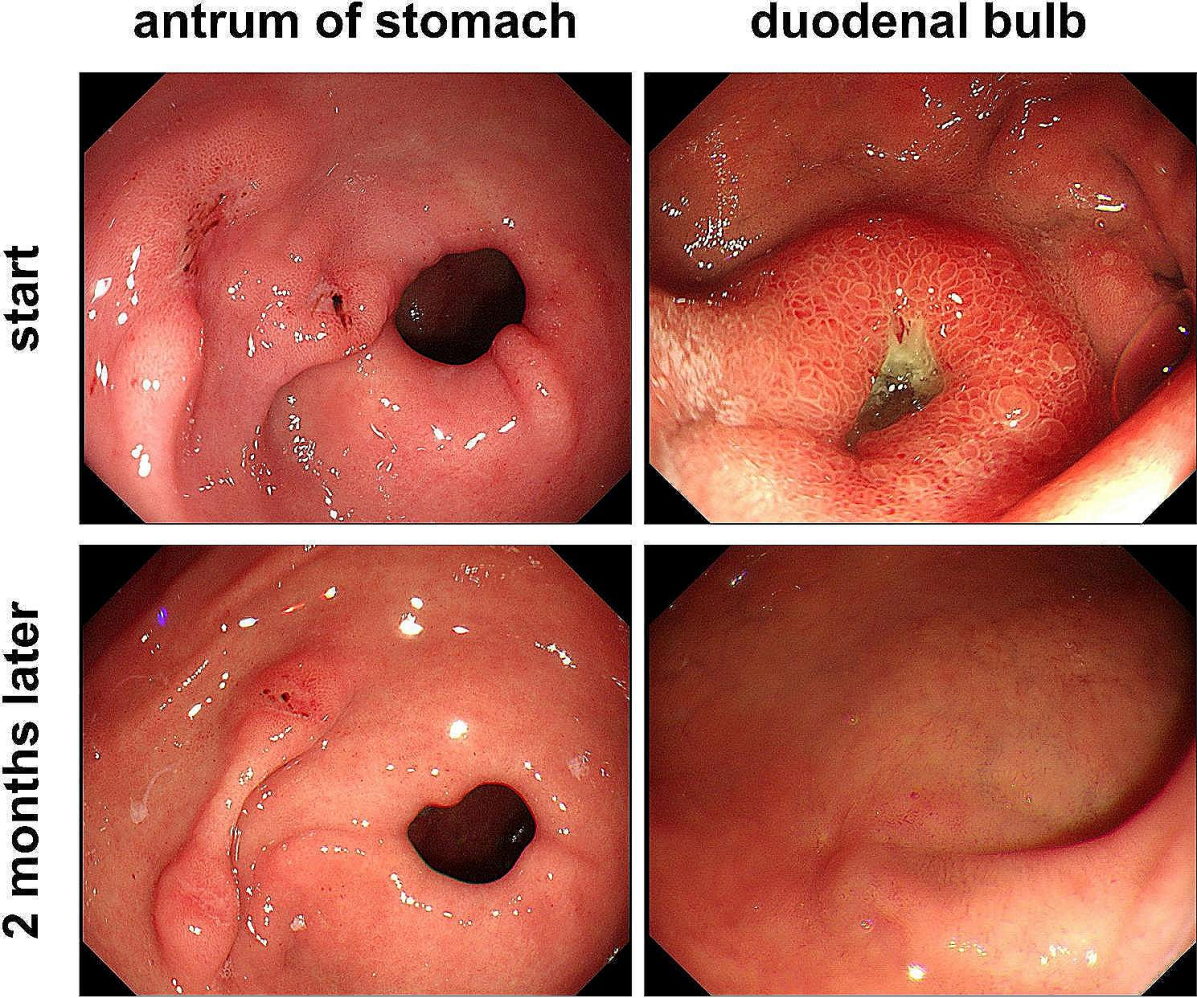
Taking omeprazole once every other day can effectively repair gastric mucosa injury. Two patients taking aspirin were found to have gastric and duodenal ulcers during pre-enrolment gastroscopy. After 2 months of treatment with omeprazole (20 mg/tablet, one tablet every other day), gastroscopy showed a significant improvement in mucosal damage

Two patients with NSAID-related peptic ulcers caused by aspirin (100 mg/d) were treated with a standard dose of omeprazole once every other day for 2 months (once every two days, 20 mg/time). One patient had 2 ulcers on the anterior wall of the gastric antrum before treatment. After 2 months of treatment, a follow-up gastroscopy showed that the ulcer near the pylorus had completely healed, and the other ulcer located on the anterior wall of the gastric antrum had shrunk from a length of approximately 8 mm before treatment to a length of approximately 2 mm after treatment. Another patient had a 5*8 mm ulcer on the anterior wall of the duodenal bulb before treatment. After 2 months of treatment, a follow-up gastroscopy showed that the ulcer had healed into a red linear scar, and red regenerative epithelium was visible around the surrounding mucosa(Fig. 6). The rapid urease test of the gastric mucosa was carried out in both patients during the first gastroscopy, and the results were negative.

## Discussion

NSAIDs are widely used in the clinic, and the representative drug is aspirin. However, NSAIDs are associated with many adverse reactions, especially peptic ulcers, bleeding, and even death. At present, PPIs are the first-line drugs for the prevention and treatment of GI adverse reactions associated with NSAIDs and have been shown to be more effective than histamine-2 receptor antagonists in repairing GI mucosal damage [11, 12]. Recent studies have shown that long-term use of PPI drugs may lead to fundic gland polyps and even GI malignancies. Studies have shown that these factors are closely related to hypergastrinemia [13].

In response to acid suppression by PPIs, antral G-cells increase gastrin production in an attempt to promote acid secretion. Gastrin is the main hormone involved in stimulating gastric acid secretion but is also a potent growth factor and facilitates both proliferative and trophic effects on the GI mucosa, particularly on the enterochromaffin-like (ECL) cells of the stomach [14]. Helgadottir, H et al. showed that the level of gastrin is closely related to the PPI dosage, where higher doses of PPIs are associated with higher gastrin levels [15]. Therefore, it is speculated that reducing the PPI dosage can reduce gastrin levels and thereby reduce potential PPI risks. However, the question that arises is, can small doses of PPIs still effectively prevent or treat peptic ulcers? Hyun Lim et al. studied the use of half the standard PPI dose to prevent upper GI bleeding in patients with chronic renal failure undergoing dialysis. The results showed that the incidence and risk of upper GI bleeding were significantly reduced in the prevention group compared to the nonprevention group (5.4% vs. 1.4%) [6]. This study to some extent indicated that half the conventional dose of PPI could still effectively prevent upper gastrointestinal bleeding, which was consistent with our study. Our research showed that taking a standard dose of omeprazole once every other day not only effectively prevented aspirin-induced gastric ulcer bleeding but also reduced the stimulatory effect on gastrin, prevented hypergastrinemia, and reduced the risk of abnormal proliferation of cells such as ECL cells.

This study revealed that there was no significant difference between omeprazole taken once a day and that taken once every other day in terms of repairing the gastric mucosa, inhibiting gastric acid secretion and reducing cell apoptosis. However, omeprazole taken once every three days had a weak ability to repair the gastric mucosa, so it should not be used as a drug regimen to prevent gastric mucosal damage caused by aspirin.

The level of gastrin in the omeprazole once a day group increased significantly compared with that in the other groups, which was consistent with the results of the clinical observation. In almost all cases, long-term use of PPIs results in hypergastrinemia, with a 3- to 5-fold increase in fasting serum gastrin levels in most individuals and much higher levels in 10–30% of cases [14]. Studies have shown that the incidence rate of gastric carcinoids from ECL cells has significantly increased over the past 30 years, which is considered related to hypergastrinemia caused by long-term PPI use, thus stimulating the proliferation, dysplasia and neoplasia of ECL cells [16, 17]. The growth-promoting effect of gastrin on ECL cells has no fixed threshold but is concentration-dependent and related to the exposure time [18]. The effect is mediated by the gastrin receptor CCK2R on the ECL cell membrane [19–21].

CCK2R is not only expressed in ECL cells and promotes heterogeneous proliferation but is also highly expressed in a variety of tumour tissues, such as pancreatic cancer, colorectal cancer, medullary thyroid cancer, etc. At present, CCK2R has become a target for radiotherapy of tumours, and radiopharmaceuticals that can effectively bind to CCK2R are being studied to target tumours and improve the effectiveness of radiotherapy [22]. The latest research aims to combine radioactive drugs with minigastrin analogues, which could efficiently bind to CCK2R to significantly improve tumour-targeting properties [23]. Our study showed that omeprazole taken once every other day not only reduces gastrin levels but also decreases CCK2R expression. Therefore, we speculate that compared with rats taking omeprazole once a day, rats taking omeprazole once every other day have lower gastrin levels and CCK2R expression, which may reduce the occurrence of gastric polyps, the proliferation of ECL cells, and even the occurrence of gastrinoma to some extent. Moreover, in this study, patients with aspirin-induced peptic ulcers were given a standard dose of omeprazole once every other day. After 2 months of treatment, gastroscopy revealed that their ulcers had significantly healed. The sample size of the clinical cases involved in this study was small (2 cases), and the purpose of administering medicine to these patients (to treat ulcers) was different from the experimental purpose (to prevent ulcers); therefore, it cannot be concluded that the standard dose of omeprazole taken by clinical patients every other day can effectively prevent aspirin-related peptic ulcers. In the follow-up, we will conduct relevant human studies. In this study, although the serum gastrin level increased after the rats were treated with omeprazole once a day, it was lower than previously reported, which was considered related to the short duration of administration. The limitation of this study is that the effects of omeprazole once every other day on the prevention of ulcers, gastrin levels, and cell proliferation were observed over a relatively short period. Further research is needed to understand the effects of long-term low-dose omeprazole. Notably, the PPI drug used in this study was omeprazole, and whether other PPI drugs have similar preventive effects remains to be further studied.

## Conclusions

In summary, omeprazole taken once every other day not only effectively prevents aspirin-induced gastric ulcers and reduces cell apoptosis but also significantly reduces serum gastrin levels and CCK2R expression, thus presumably reducing the occurrence of gastric polyps and possibly gastric cancer induced by long-term administration of PPIs.

### Electronic supplementary material

Below is the link to the electronic supplementary material.

Supplementary Material 1

Supplementary Material 2

## Data Availability

All data generated or analysed during this study are included in this published article and its supplementary information files, there are no additional unpublished data. You may contact [corresponding author/bhxu22@ccmu.edu.cn] with data requests, and the data will be provided upon reasonable request.

## Abbreviations

PPIs: proton-pump inhibitors
NSAIDs: nonsteroidal anti-inflammatory drugs
qd: once every day
qod: once every other day
1/d3: once every three days
GI: gastrointestinal
ECL: intestinal chromaffin cell
SD: Sprague‒Dawley
Ome: omeprazole
CCK2R: cholecystokinin B receptor

## References

[1] Kleindorfer DO, Towfighi A, Chaturvedi S et al. Guideline for the Prevention of Stroke in Patients With Stroke and Transient Ischemic Attack: A Guideline From the American Heart Association/American Stroke Association. Stroke. 2021;52:e364–467 10.1161/STR.0000000000000375 34024117

[2] Yeomans ND, Lanas AI, Talley NJ (2005). Prevalence and incidence of gastroduodenal ulcers during treatment with vascular protective doses of aspirin. Aliment Pharm Ther.

[3] Moayyedi P, Eikelboom JW, Bosch J (2019). Safety of Proton Pump inhibitors based on a Large, multi-year, randomized trial of patients receiving Rivaroxaban or Aspirin. Gastroenterology.

[4] Freedberg DE, Kim LS, Yang YX (2017). The risks and benefits of long-term use of Proton Pump inhibitors: Expert Review and best practice advice from the American Gastroenterological Association. Gastroenterology.

[5] Ally MR, Veerappan GR, Maydonovitch CL (2009). Chronic proton pump inhibitor therapy associated with increased development of fundic gland polyps. Digest Dis Sci.

[6] Lim H, Kim JH, Baik GH (2015). Effect of low-dose proton pump inhibitor on preventing upper gastrointestinal bleeding in chronic kidney disease patients receiving aspirin. J Gastroen Hepatol.

[7] Al-Quraishy S, Othman MS, Dkhil MA, Abdel MA (2017). Olive (Olea europaea) leaf methanolic extract prevents HCl/ethanol-induced gastritis in rats by attenuating inflammation and augmenting antioxidant enzyme activities. Biomed Pharmacother.

[8] Fan DD, Lin S, Song YP (2016). Astragaloside IV protects rat gastric mucosa against aspirin-induced damage. Int Immunopharmacol.

[9] Nam SY, Kim N, Lee CS (2005). Gastric mucosal protection via enhancement of MUC5AC and MUC6 by geranylgeranylacetone. Digest Dis Sci.

[10] Weng J, Wang X, Xu B, Li W (2021). Augmenter of liver regeneration ameliorates ischemia-reperfusion injury in steatotic liver via inhibition of the TLR4/NF-kappaB pathway. Exp Ther Med.

[11] Scally B, Emberson JR, Spata E (2018). Effects of gastroprotectant drugs for the prevention and treatment of peptic ulcer disease and its complications: a meta-analysis of randomised trials. Lancet Gastroenterol.

[12] Kamada T, Satoh K, Itoh T (2021). Evidence-based clinical practice guidelines for peptic ulcer disease 2020. J Gastroenterol.

[13] Hongo M, Fujimoto K (2010). Incidence and risk factor of fundic gland polyp and hyperplastic polyp in long-term proton pump inhibitor therapy: a prospective study in Japan. J Gastroenterol.

[14] Lee L, Ramos-Alvarez I, Ito T, Jensen RT. Insights into Effects/Risks of chronic hypergastrinemia and lifelong PPI treatment in Man based on studies of patients with Zollinger-Ellison Syndrome. Int J Mol Sci. 2019; 20. 10.3390/ijms20205128 PMC6829234 31623145

[15] Helgadottir H, Lund SH, Gizurarson S, Metz DC, Bjornsson ES (2020). Predictors of Gastrin Elevation Following Proton pump inhibitor therapy. J Clin Gastroenterol.

[16] McCarthy DM. Proton Pump inhibitor Use, Hypergastrinemia, and gastric carcinoids-what is the relationship? Int J Mol Sci. 2020;21. 10.3390/ijms21020662 PMC7014182 31963924

[17] Kidd M, Gustafsson B, Modlin IM (2013). Gastric carcinoids (neuroendocrine neoplasms). Gastroenterol Clin N.

[18] Lundell L, Vieth M, Gibson F, Nagy P, Kahrilas PJ (2015). Systematic review: the effects of long-term proton pump inhibitor use on serum gastrin levels and gastric histology. Aliment Pharm Ther.

[19] Sheng W, Malagola E, Nienhuser H (2020). Hypergastrinemia expands gastric ECL cells through CCK2R(+) progenitor cells via ERK Activation. Cell Mol Gastroenter.

[20] Brenna E, Waldum HL (1992). Trophic effect of gastrin on the enterochromaffin like cells of the rat stomach: establishment of a dose response relationship. Gut.

[21] Fossmark R, Martinsen TC, Waldum HL. Adverse effects of Proton Pump inhibitors-evidence and plausibility. Int J Mol Sci. 2019; 20. 10.3390/ijms20205203 PMC6829383 31640115

[22] von Guggenberg E, Kolenc P, Rottenburger C, Mikolajczak R, Hubalewska-Dydejczyk A. Update on Preclinical Development and clinical translation of Cholecystokinin-2 receptor targeting Radiopharmaceuticals. Cancers. 2021; 13. 10.3390/cancers13225776 PMC8616406 34830930

[23] Hormann AA, Klingler M, Rangger C et al. Effect of N-Terminal peptide modifications on in Vitro and in vivo Properties of (177)Lu-Labeled peptide analogs targeting CCK2R. Pharmaceutics. 2023;15. 10.3390/pharmaceutics15030796 PMC10058949 36986657

