# Supplementary figures and images for: Omeprazole taken once every other day can effectively prevent aspirin-induced gastrointestinal mucosal damage in rats
Source: BMC Gastroenterol. 2024 May 29;24:187. doi: 10.1186/s12876-024-03265-0 (PMC11134753; doi:10.1186/s12876-024-03265-0)

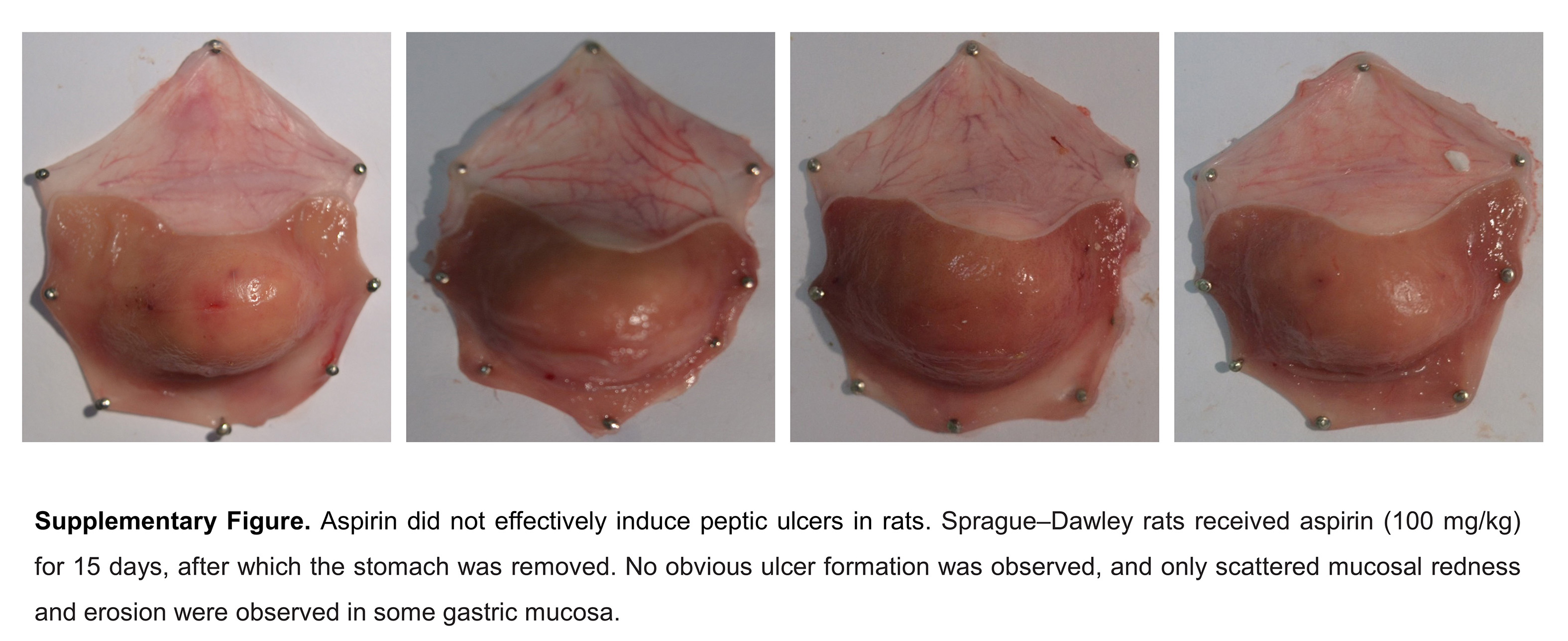

Supplement: Supplementary file 1 — Supplementary Material 1 [file 12876_2024_3265_MOESM1_ESM.jpg]
