# Supplementary material for: Omeprazole taken once every other day can effectively prevent aspirin-induced gastrointestinal mucosal damage in rats
Source: BMC Gastroenterol. 2024 May 29;24:187. doi: 10.1186/s12876-024-03265-0 (PMC11134753; doi:10.1186/s12876-024-03265-0)
Supplement: Supplementary file 2 — Supplementary Material 2 [file 12876_2024_3265_MOESM2_ESM.docx]

We conducted a preliminary experiment before determining the final concentration of aspirin and found that the continuous administration of aspirin (100 mg/kg/d) to Sprague‒Dawley rats for 15 days did not effectively induce the occurrence of peptic ulcers.

Male Sprague‒Dawley rats (250‒300 g) aged 8 weeks were purchased from Shanghai SLAC Laboratory Animal Co., Ltd. (Shanghai, China), housed in hygienic cages at 22‒24°C with a 12-h cycle of darkness and light and had free access to water and food. After a 1-week adaptation period, six rats received aspirin (100 mg/kg) through oral gavage once a day at 9:00 am for 15 days. At the end of the experiment, after 10 hours of fasting, the rats were sacrificed with carbon dioxide. After blood samples were collected from the abdominal aorta, the stomach was removed and fixed with needles.

No obvious ulcer formation was observed in the gastric mucosa of the rats. Scattered mucosal redness, swelling and erosion could be seen in some gastric mucosa, while no obvious damage was found in some gastric mucosa.
